# Unusual linkage patterns of ligands and their cognate receptors indicate a novel reason for non-random gene order in the human genome

**DOI:** 10.1186/1471-2148-5-62

**Published:** 2005-11-08

**Authors:** Laurence D Hurst, Martin J Lercher

**Affiliations:** 1 Department of Biology and Biochemistry, University of Bath, Bath, BA2 7AY, UK

## Abstract

**Background:**

Prior to the sequencing of the human genome it was typically assumed that, tandem duplication aside, gene order is for the most part random. Numerous observers, however, highlighted instances in which a ligand was linked to one of its cognate receptors, with some authors suggesting that this may be a general and/or functionally important pattern, possibly associated with recombination modification between epistatically interacting loci. Here we ask whether ligands are more closely linked to their receptors than expected by chance.

**Results:**

We find no evidence that ligands are linked to their receptors more closely than expected by chance. However, in the human genome there are approximately twice as many co-occurrences of ligand and receptor on the same human chromosome as expected by chance. Although a weak effect, the latter might be consistent with a past history of block duplication. Successful duplication of some ligands, we hypothesise, is more likely if the cognate receptor is duplicated at the same time, so ensuring appropriate titres of the two products.

**Conclusion:**

While there is an excess of ligands and their receptors on the same human chromosome, this cannot be accounted for by classical models of non-random gene order, as the linkage of ligands/receptors is no closer than expected by chance. Alternative hypotheses for non-random gene order are hence worth considering.

## Background

One of the most striking discoveries in the post-genomic age has been the amount of non-random gene positioning in eukaryotic genomes [1]. In the human genome, for instance, highly/broadly expressed genes cluster [2-5]. Likewise in yeast co-expressed genes tend to reside together [6] and such pairs tend also to be retained together over evolutionary time more than expected, given the intergene distance between them [7]. Blocks of broadly expressed mammalian genes also seem to be preserved over evolutionary time more than expected [8]. In *Caenorhabditis *[9-11], *Drosophila *[12-14] and *Arabidopsis *[15], to name but three, there exists further evidence for expression clusters of some variety. These results all suggest that eukaryotic genomes are organised in a manner that permits co-expression or co-ordinate expression. Evidence also suggests linkage of functionally related genes, although on this issue the evidence is more equivocal, not least because of an ambiguity as to what "functionally related" can mean. On the one hand, in numerous eukaryotic genomes, genes from the same metabolic pathway cluster more than expected by chance [16] (for detailed case history see [17]). Likewise, linked co-expressed genes in yeast often fall within the same MIPs (Munich Information Centre For Protein Sequences) category [6] or the same Gene Ontology (GO) classification [18].

These results are so striking because they so profoundly overturn the long held assumption that genes are randomly located around eukaryotic genomes. This is not to say that possible exceptions were not considered prior to the sequencing of the complete genome. They were, however, typically dismissed as being unrepresentative or uninteresting either because they were clearly the product of tandem duplication (hox cluster, globin cluster) or were associated with weird genetics (imprinted clusters) or genes that are otherwise exceptional (e.g. clustering of rRNAs). Not all such suggestive examples could so easily be dismissed however. Here we concentrate on one class, linkage of ligands to their cognate receptors. This issue is worth systematic analysis, not least because in yeast it has recently been shown that genes whose proteins interact to form stable complexes are linked more often than expected by chance [19].

That ligands and their receptors may be linked was observed independently by several workers. Cooper [20], noting that the linkage of ligands to receptors may be common, highlights the examples of transferrin and transferrin receptor on chromosome 3q, as well as apolipoprotein E and the low density lipoprotein receptor both on chromosome 19. He also rightly cautions, however, than one can find numerous cases where ligands and receptors are not linked. Similarly, Lennard et al. [21] note the linkage of the three ligands in the interleukin 1 cluster (IL1 alpha, beta and receptor antagonist) to the two receptors [21]. The linkage of ligands to receptors has even proven to have some predictive power. Wang et al. [22] noticed that hepatocyte growth factor (HGF) and its MET receptor were both on 7q. Noting too the presence in 3p21 of both macrophage stimulating factor (MST1, a member of the same gene family as HGF), and RON (a member of the MET receptor family), they hypothesised that RON might be MST1's receptor [22]. This, in turn, they demonstrated to be the case (RON's alias is now MST1R) [22]. Popovici et al. note several of the above examples and also point to a total of 14 incidences of linked genes involved in the same pathway, not necessarily as ligand-receptor couplings [23].

This evidence prompts two questions. First, is it true that there is something odd about the linkage patterns of ligands and their cognate receptors? Second, if it is true, why might this be so? Prior authors have also suggested that linkage of ligands to receptors might be functionally important. Haig [24], observing two of the above cases (interleukin 1 and transferrin), notes that close proximity could enable linkage disequilibrium between alleles at the ligands and receptors. This linkage disequilibrium would potentially enable the spread of rare allele combinations for which there exist particular epistatic interactions. These may, Haig suggests, act as selfish maternal effect lethals, an example of which has been described in mice [25,26]. This theory may be seen as a special case of a more general theory for linkage based on preservation of linkage disequilibrium under epistasis [27-30]. One might also conjecture that ligands and receptors might at times need to be co-expressed [see e.g. [31]], so very close linkage might be beneficial for this reason as well.

If selection does act on the location of ligands and receptors (either to permit co-expression or to maintain linkage disequilibrium), then we should predict, from the above models, that when two such genes are on the same chromosome they should also be, on average, physically closer than would be expected by chance. To this end we ask two questions. First, is the mean distance between ligands and their linked receptors shorter than expected by chance? As this mode of analysis could miss an excess of cases with very tight linkage, we additionally ask whether the number of incidences of linkage within a given window size (1 Mb, 2 Mb etc.) is higher than expected by chance.

## Results and discussion

### No evidence for close proximity of ligands and receptors

If ligands and their cognate receptors were under selection to be in close physical proximity, we should find that the mean distance between them should be smaller than expected by chance. To test this, we examined ligand-receptor pairs from the DLRP database [32,33]. All analyses were also performed for an augmented dataset, which additionally contains two 'cherry-picked' cases highlighted by Cooper [20] (see Methods). Contrary to expectations, the mean distance between ligand and receptor in the non-augmented data set (64.579 Mb) is higher than that found in randomized genomes (56.344 Mb; *P *= 0.733). The same pattern is found in the augmented data set (real = 63.466 Mb, randomized = 56.165 Mb, *P *= 0.709). Note that a ligand can have many receptors and that this is factored into the analysis through the randomization protocol.

It may, however, be the case that there exist a number of ligand-receptor pairs that are much closer to each other than expected. To examine this we compared the number of ligand-receptor pairs within some critical distance of each other and compared this with the number expected by chance. In these simulations we permuted genes only within the chromosomes within which they are found, so as to ensure that the number of ligand-receptor pairs were the same in the randomized sets as in the real data set. As can be seen (Table 2) in neither data set do we find evidence for anything other than a pattern of random linkage [see Additional file 4].

**Table 2:** The number of occurrences of a ligand receptor pair within a given distance of each other (in Mb) for the real genome compared with 10000 randomized genomes. In this instance the first three results columns refer to the dataset excluding the two examples identified by Cooper.

**Distance (Mb)**	**Obs**	**Exp**	**P**	**Obs**	**Exp**	**P**
0.2	0	0	1	0	0	1
0.5	1	0.09	0.095	1	0.09	0.087
1	1	0.17	0.163	1	0.16	0.152
2	1	0.94	0.672	1	0.94	0.67
5	1	1.62	0.864	1	1.59	0.855
10	2	3.18	0.927	2	3.16	0.917
20	7	6.20	0.413	7	6.18	0.409
50	12	10.94	0.363	13	12.0	0.385

It has been previously established that broadly expressed genes cluster [3,4]. Might our simulations have produced misleading results by permitting genes of the ligands and receptors to reside in any chromosomal location? To examine this possibility we considered randomizations in which genes are swapped exclusively with ones of the same breadth of expression. None of the above results are qualitatively affected (Table 3). Using a different bin size to classify breadth of expression appears to have no effect on the results (Table 3). Likewise, permitting ligands and receptors to be located in the genome at the locations of other ligands and receptors does not affect any conclusions [see Additional file 4].

**Table 3:** Results of randomizations controlling for breadth of expression. The "Aug" (Augmented) data set is that containing the two ligand -receptor sets nominated by Cooper [20]. Bin size indicates the span of breadths of expression considered to be the same in the randomizations. Bin size one implies that only genes of the same breadth were switched with each other. Distances are measured in Mb. *P*-values are estimated by comparison of observed data with expectations obtained from randomised genomes.

**Data set**	**Bin size**	**N**	**# on same chr (observed)**	**# on same chr (expected)**	***P***	**Mean dist. (observed)**	**Mean dist. (expected)**	***P***
Aug	1	267	25	14.18	0.011	63.47	55.22	0.741
Aug	5	267	25	14.14	0.01	63.47	55.77	0.705
Non-Aug	1	265	23	14.09	0.027	64.58	56.25	0.731
Non-Aug	5	265	23	13.8	0.013	64.58	55.47	0.752

The above results indicate that there is no evidence for selection for clustering of ligand and receptor. For this reason we reject a model positing epistasis between alleles of ligand and receptor as a general force acting on genomic location of these genes. Moreover the lack of tight clustering suggests that we are not witnessing clustering to enable co-regulation (by ensuring that genes are co-localised in the same chromatin block). The model suggested by Haig [24] is not, however, necessarily falsified by the above results, as he postulates selection on disequilibrium only if the genes might be involved in maternal-foetal interactions. Such a model is hard to falsify in the absence of segregation/viability data from appropriate haplotypes. However, we can note that if we further restrict our data sets to those in which either the ligand or one of the receptors is placentally expressed, the qualitative patterns described above are unaltered [see Additional file 4]. We find, therefore, no evidence for close linkage of ligand and receptor when involvement in maternal-foetal interactions might be a possibility.

### An excess of ligand-receptor pairs on the same human chromosome

Above we asked whether ligands and their receptors are more closely linked than expected by chance. We can also ask if ligands and their receptors are more commonly linked (i.e. on the same chromosome) than expected by chance? In an unbiased unaugmented human data set (i.e. without the addition of the two sets highlighted by Cooper [20], see Methods) we observe 23 such pairings but expect on average 13.71 (*P *= 0.015). When we include the two extra sets the *P *value, as expected, is reduced: we observe 25 pairs but expect on average 13.8 (*P *= 0.005). These results support the view that in the human genome linkage of a ligand to at least one of its cognate receptors is more common than would be expected by chance. However, the majority (approx 78%) of ligands are not linked to any of their receptors, so this excess should not be considered a strong rule (although, as already noted, in special cases it has had predictive power).

### No evidence for an excess of ligands-receptor pairs on the same chromosome in mouse

To ask whether the patterns observed in the human genome are also found in the mouse genome we constructed three mouse data sets and applied the three randomization protocols to each. The first two data sets are the ortholog equivalents of our two human data sets purged of duplicates by either a) Blasting or b) Blasting and removal by physical proximity (in the human genome) of ligands or receptors. That is, if two ligands were in close proximity in the human genome, even if not identified as sequence related, we would remove one before considering the location of the mouse orthologs. However, as it is possible that some ligand clusters might be unique to mouse, we additionally purged the more stringent of the above two of any groupings of ligands or receptors seen in the mouse genome.

As it happens, no matter which data set one employs or which randomization method is performed, there is not even a remote hint that ligands and cognate receptors occur more commonly on the same mouse chromosome than expected by chance [see Additional file 5]. For example, in the equivalent of the human data set purged of duplicates by Blast alone, we observe 19 ligand-receptor pairs on the same chromosome and expect 19.38 (*P *= 0.56) in a randomization in which the ligands and receptors can assume any genomic position currently associated with a gene. In this analysis the mean distance between ligand and receptor is 57.3 Mb but is 43.4 in the randomizations (*P *= 0.937). At no specified distance do we find more pairs than expected by chance [see Additional file 5]. Controlling for breadth of expression makes no difference to this conclusion [see Additional file 6]. The list of linked genes from the data set equivalent to the human set presented in Table 1 is presented in Table 4. In this set 16 ligand-receptor pairs co-occur on the same chromosome, with 14.5 expected. Only four ligand-receptor pairs are in common in the two comparable data sets.

**Table 1:** Incidences of occurrence, on the same human chromosome, of a ligand with one of its cognate receptors after removal of tandem duplicates by Blast and by the physical proximity method. The distance is defined as the span between the mid-positions of the ligand and the mid-position of the receptor.

**Class**	**Gene**	**Unigene #**	**Cytogenetic**	**Distance (Mb)**
Ligand	*DLL1*	368657	6q27	
Receptor	*NOTCH4*	436100	6p21.3	138.23
				
Ligand	*DLL3*	127792	19q13	
Receptor	*NOTCH3*	8546	19p13.2-p13.1	29.53
				
Ligand	*EFNA4*	449913	1q21-q22	
Receptor	*EPHA8*	283613	1p36.12	129.2
				
Ligand	*TNFSF18*	248197	1q23	
Receptor	*TNFRSF18*	212680	1p36.3	168.57
				
Ligand	*IL1RN*	81134	2q14.2	
Receptor	*IL1R2*	25333	2q12-q22	11.51
				
Ligand	*HGF*	396530	7q21.1	
Receptor	*MET*	419124	7q31	34.96
				
Ligand	*MST1*	349110	3p21	
Receptor	*MST1R*	2942	3p21.3	0.21
				
Ligand	*FGF1*	278954	5q31	
Receptor	*FGFR4*	165950	5q35.1-qter	34.45
				
Ligand	*FGF2*	422889	4q26-q27	
Receptor	*FGFR3*	1420	4p16.3	122.37
				
Ligand	*FGF8*	57710	10q24	
Receptor	*FGFR2*	404081	10q26	19.77
				
Ligand	*FGF10*	248049	5p13-p12	
Receptor	*FGFR4*	165950	5q35.1-qter	132.07
				
Ligand	*FGF17*	248192	8p21	
Receptor	*FGFR1*	748	8p11.2-p11.1	16.46
				
Ligand	*FGF18*	87191	5q34	
Receptor	*FGFR4*	165950	5q35.1-qter	5.65
				
Ligand	*VEGFC*	79141	4q34.1-q34.3	
Receptor	*KDR*	12337	4q11-q12	122.23
				
Ligand	*PTN*	44	7q33-q34	
Receptor	*PTPRZ1*	78867	7q31.3	15.23
				
Ligand	*TGFB2*	169300	1q41	
Receptor	*TGFBR3*	342874	1p33-p32	122.99
				
Ligand	*BMP3*	121507	4p14-q21	
Receptor	*BMPR1B*	480321	4q22-q24	13.97
				
Ligand	*BMP10*	158317	2p13.3	
Receptor	*ACVR1*	150402	2q23-q24	89.45
Receptor	*ACVR2*	283349	2q22.2-q23.3	79.47
				
Ligand	*INHBB*	1735	2cen-q13	
Receptor	*ACVR1*	150402	2q23-q24	37.64
Receptor	*ACVR2*	283349	2q22.2-q23.3	27.66
				
Ligand	*INHA*	407506	2q33-q36	
Receptor	*ACVR1*	150402	2q23-q24	61.8
Receptor	*ACVR2*	283349	2q22.2-q23.3	71.79
				
Ligand	*TF*	433923	3q22.1	
Receptor	*TFRC*	185726	3q29	62.32
				
Ligand	*APOE*	110675	19q13.2	
Receptor	*LDLR*	213289	19p13.3	39.02

**Table 4:** Incidences of occurrence, on the same mouse chromosome, of a ligand with one of its cognate receptors after removal of tandem duplicates by Blast and position methods. The distance is defined as the span between the mid-positions of the ligand and the mid-position of the receptor. The receptors indicated with a Y in the conserved linkage column are those that are also on the same chromosome as the same ligand in the comparable human genome set (i.e. those in Table 1).

**Class**	**Gene**	**Unigene #**	**Chromosome**	**Distance (Mb)**	**Conserved linkage**
Ligand	*Jag1*	Mm.22398	2		
Receptor	*Notch1*	Mm.290610	2	110.41	
					
Ligand	*Dll4*	Mm.143719	2		
Receptor	*Notch1*	Mm.290610	2	92.67	
					
Ligand	*Bmp2*	Mm.103205	2		
Receptor	*Acvr1*	Mm.689	2	74.85	
Receptor	*Acvr2*	Mm.314338	2	84.49	
					
Ligand	*Bmp7*	Mm.595	2		
Receptor	*Acvr1*	Mm.689	2	114.51	
Receptor	*Acvr2*	Mm.314338	2	124.15	
					
Ligand	*Tnfsf8*	Mm.4664	4		
Receptor	*Tnfrsf8*	Mm.12810	4	81.45	
					
Ligand	*Pdgfa*	Mm.2675	5		
Receptor	*Pdgfra*	Mm.221403	5	62.52	
					
Ligand	*Ptn*	Mm.279690	6		
Receptor	*Ptprz1*	Mm.41639	6	13.86	Y
					
Ligand	*Fgf15*	Mm.3904	7		
Receptor	*Fgfr2*	Mm.16340	7	14.87	
					
Ligand	*Mst1*	Mm.8369	9		
Receptor	*Mst1r*	Mm.3901	9	0.17	Y
					
Ligand	*Ifng*	Mm.240327	10		
Receptor	*Ifngr1*	Mm.549	10	98.83	
					
Ligand	*Fgf10*	Mm.317323	13		
Receptor	*Fgfr4*	Mm.276715	13	61.47	Y
					
Ligand	*Bmp4*	Mm.6813	14		
Receptor	*Bmpr1a*	Mm.237825	14	8.55	
					
Ligand	*Dll1*	Mm.4875	17		
Receptor	*Notch3*	Mm.4945	17	16.56	
Receptor	*Notch4*	Mm.173813	17	18.88	Y

### Explaining the data: interesting biology or statistical artefact?

We find no evidence in mouse or man that ligands and their receptors are more closely linked on average than expected by chance. However, we do find that there are more ligand-receptor pairs on the same human chromosome than expected, a feature not found in mouse. From the above results we are faced with two possible explanations for the human data. First, that it is just a statistical blip, possibly owing to some subtle bias in the original data set (note for example, that MST1R was analysed as a potential receptor because of its linkage to MST [22]). Second, that the excess of ligand-receptor pairs on the same chromosome is the product of some deterministic force, that for some reason does not apply, or is not strong enough, in rodents. This might either be direct selection favouring the persistence of co-occurrence or a deterministic bias in the creation of co-occurrence.

That the pattern is found in humans rather than mice argues against a direct selective benefit for co-retention on the same chromosome. This is owing to the fact that the effective population size of the human population is most probably much smaller than that of mice. As such, according to the nearly-neutral theory, the efficacy of selection should be higher in mice (see also [34,35]). Hence, if the pattern was owing to selection directly favouring co-occurrence, it is more likely to be observable in mouse rather than human, all else being equal. Moreover, it is also hard to see what direct selective benefit might accrue from co-occurrence in weak linkage. In particular, co-regulation of ligands and receptors (which is not clearly expected in the first place) would likely require much tighter linkage than observed here. We also find no evidence for a stronger similarity in the breadth of expression of the linked ligands and receptors than those on different chromosomes. We considered the difference in breadth of expression between ligand and receptor normalised by the mean of the two. Linked genes are of no more similar breadth of expression (mean difference for linked genes 0.75 +/- 0.12, for unlinked 0.686 +/- 0.036, t-test, P = 0.58).

### Segmental duplication and the balance hypothesis: an alternative hypothesis for non-random gene order

A notable feature of our data set is that there are numerous cases in which a ligand-receptor pair in linkage is matched by at least one other paralogous pair also in linkage. If we define genes belonging to the same Hovergen [36] family as paralogs, then we can identify the following linked paralogous pairs from Table 1: HGF/MST1 (ligands) with MET/RON (receptors); DLL1/DLL3 with NOTCH3/NOTCH4; FGF1/FGF2 with FGFR3/FGFR4; FGF8/FGF17 with FGFR1/FGFR2 (see also [23]). Note too that FGF18 is linked to FGFR4 and to FGF1 and is sequence related to FGF8 and FGF17.

It has been argued that co-paralogy of gene pairs involved in the same pathway (of which ligand-receptor pairs are but one example) appear to be unusually common [23]. This finding is also in accord with much recent evidence suggesting that the human genome (and the vertebrate genome more generally) may be a mosaic of old large block duplications (i.e., duplications of large chunks of DNA sequence) and/or the result of whole genome duplications [37], with several of the above paralogous groups being claimed to be the result of such duplication events: FGFR 1, 2, 3 and 4 are in paralog clusters on human chromosomes 8p, 10q, 4p, and 5q respectively [38]; NOTCH 3 and 4 also appear to be in paralog clusters on chromosomes 6 and 19, with NOTCH 1 and NOTCH 2 being two further duplications of the same block [39]; HGF/MST1 and MET/RON were also previously described as belonging to co-paralogous groups [23].

Following an earlier hypothesis [19], we would like to suggest an hypothesis to explain our results that is based on the occurrence of block duplications [40]. If some ligands and receptors require an appropriate balance in their titres, then one could expect that a mutation resulting in a block duplication containing one of the pair (e.g., the ligand) might be more likely to spread through the population if it also duplicates the other (e.g., the receptor). Such co-duplication is most likely if ligand and receptor happen to be linked, while unlinked ligand-receptor pairs are less likely to be successfully duplicated. Our hypothesis may be considered as being a form of the balance hypothesis [41,42], which supposes that proteins involved in mutual interactions need to have their titres appropriately balanced. Direct evidence for this proposition has been described in yeast, in which it is also reported that the need for balance might explain the lack of duplicability of the genes involved in complex formation [43].

This hypothesis appears tenable in the current context for several reasons. It is, for example, suggestive that the paralogous pair sets tend to be more closely linked, although the statistic is on the edge of significance (Median Test, ChiSquared = 3.59, *P *= 0.058, df = 1). This would be expected if constraints exist on the upper size limit of the block duplications. It may also be notable that for the many of the above genes there is evidence for dosage sensitivity, as required by the balance hypothesis (Table 5). Moreover, as re-arrangements tends to be especially common in mice [44-46], any linked pairs possibly generated by block duplication are more likely to be split up, making the genome more like random.

**Table 5:** Evidence for or against dosage sensitivity of ligand and receptors that were possibly the source for or the consequence of block duplication

**Gene**	**Evidence for dose sensitivity**	**References**
*HGF*	Over-expression in retinal pigment epithelium induces retinal detachment	[58]
MET*	Autosomal dominant Hereditary papillary renal carcinoma is associated with mutations in MET	[59]
*MST1*	None: mouse knockout is without strong phenotype	[60]
*RON*	Hemizygous mice (Ron +/-) are highly susceptible to endotoxic shock and are compromised in their ability to downregulate nitric oxide production	[61]
*DLL1*	No report of heterozygous null phenotype nor of overexpression phentype	
NOTCH3*	Autosomal dominant disorder CADASIL owing to mutation in NOTCH3	[62]
*DLL3*	None: the gene is associated with disease (SCDO1/2) in mutant homozygotes but no report of heterozygote phenotype.	
*NOTCH4*	Upregulation of NOTCH4 is associated with mammary tumours	See OMIM 164951
*FGF1*	None: no phenotype in Fgf1 homozygous knockouts	[63]
FGFR3*	Autosomal dominant disorder ACH associated with mutation in FGFR3	See OMIM 134934
*FGF2*	Over-expression promotes bone growth	[64]
*FGFR4*	None: knockout homozygotes have no obvious phenotype	[65]
*FGF8*	Over-expression is associated with carcinogenesis	[66]
FGFR1*	Autosomal dominant Pfeiffer syndrome is owing to mutations in FGFR1	[67]
*FGF17*	None: heterozygote knockout has no phenotype	[68]
FGFR2*	Numerous autosomal dominant disorders associated with FGFR2	See OMIM 176943
*FGF18*	Dose sensitive liver and small intestine development	[69]

*Note: These five genes are known to be associated with human autosomal dominant disorders. Searching OMIM [70] for mapped genes with "autosomal dominant" somewhere in the title or the text and not "recessive" in the title, reveals an estimate of 1166 mapped genes which may be associated with autosomal dominant disorders. Assuming 22470 autosomal genes (as in the NC files) we should then have expected from a random sample of 17 autosomal genes less than one dominant, significantly less than observed (Chi-squared, P < 0.0001). However, in some cases the dominance is owing to negative mutations rather than dose *per se*.

The above hypothesis also predicts that ligands and their linked receptors might be duplicated at the same time. While this can be approximately established by phylogenetic methods, these do not constitute a perfect test, as they fail to establish whether the pairs were duplicated in a block together and furthermore, genes known to be co-duplicated are very commonly not identified as such by phylogenetic methods [47]. Nonetheless, we have surveyed the available data and prior analyses and fail to find any data that contradicts the hypothesis that when a given receptor duplicated the relevant ligand did as well [see Additional file 7]. The ligands FGF1 and FGF18 are, however, probably the result of an ancient duplication that occurred independent of the receptor [48].

In some of the incidences reported here, a case for co-duplication has already been made. The linkage of the FGFs to their receptors has previously been argued, from phylogenetic data, to be owing to block or whole genome duplications [48]. This view is supported by our inspection of the phylogenetic tree of the FGFR family as presented in Hovergen [36], which suggests that at the base of the vertebrates there was one receptor which duplicated to produce the ancestors of FGFR1/2 and FGFR3/4. Duplication of both ancestral sequences then occurred very shortly after (prior to the divergence of the fish), leaving FGFR1 and FGFR2 as nearest paralogs, and FRGR3 and FGFR4 as nearest paralogs. If there was co-duplication of the receptors, we should expect to see FGF1 and FGF2 as nearest paralogs and FRF8 and FGF17 as nearest paralogs, with, in both incidences, duplication occurring near the base of the vertebrates. The nearest paralog relationships are indeed upheld [48]. Furthermore, in both instances the duplication occurred prior to the divergence of fish, as predicted.

## Conclusion

In sum, we have described a novel pattern of co-localisation on the same chromosome of genes whose products interact, which cannot obviously be accounted for either by known models for co-ordinate regulation, nor by selection for linkage disequilibrium. The pattern may in part reflect a past history of block duplication. A version of the balance hypothesis is worth considering as underpinning to explain the results.

Tests of this hypothesis should be possible in the future. We should in principle be able, with fuller knowledge of gene order in many mammals, to reconstruct the past history of duplication and gene order re-arrangements that occurred through mammalian history. The model predicts an excess of block duplications in which both ligand and cognate receptor are found, as well as excess in which neither are found, but a dearth of those with one, but not the other. The model also predicts a general weakening of this initial signal with increasing numbers of inter-chromosomal re-arrangements, as the hypothesis proposes only an initial filter of block duplications, not ongoing direct selection to maintain linkage.

## Methods

### Data set assembly and curation

The table of ligand-receptor partners were extracted from the DLRP database [32,33]. This specifies for any given single ligand the corresponding receptor or set of receptors. The genes here are referred to by gene name and Unigene id number, by reference to an old release of Unigene. These entries we updated to the current release for Homo sapiens, UniGene Build #175. For each Unigene number and gene name, the relevant Unigene page was identified [49]. If the entry remained in the new build all details were left unchanged, except in three cases where there exists a gene by the same name as that in the original dataset, at the same genomic location as the given Unigene entry, but in a separate Unigene class. In these cases the Unigene entry with matching name was employed. If the old entry had been retired then a) if only one new entry is available this was used, b) if multiple entries were found (i.e. the cluster has split), then the one with the gene name identical to that of the old entry was used, c) if no entry had the same name but all entries were at the same genomic location the entry with the most abundant sequence data was used, d) if no unambiguous match could be found the entry was eliminated. If this was the ligand then the whole entry was deleted. In a few cases separate ligand receptor blocks are collapsed to the same Unigene entry in build #175 (e.g. FGFR and FGFRB). In this instance one of the two sets was eliminated. The original file also contains a number of entries in which the ligand alone is given, with no receptor. In these cases the entry was deleted.

From this new set of Unigene identities Entrez gene [50] was searched with the current Unigene id being posted. From here we recovered a) the Entrez/LocusLink gene name b) the Entrez/LocusLink id. If the LocusLink/Entrez gene name was different from the Unigene name then the pairs were examined at LocusLink to determine that the names were synonymous. In all cases this proved to be so. From here we obtained the physical location in the NC Genbank files for each chromosome. The cDNA source annotation of each Unigene entry was employed to determine whether the gene was placentally expressed.

The data set does not include the two cases highlighted by Cooper [20]: transferrin and its receptor and apolipoprotein E and its receptor. We therefore consider a second data set in which we add these two. This is, however, problematic as we are adding only "cherry picked" data. It is thus much more likely that we should find close linkage in the expanded set compared with the original set, owing to the non-random nature of the addition to the data set. Nonetheless, should we find an absence of an effect in this expanded set, this would make for stronger evidence against the hypothesis of an over-abundance of close linkage of ligands and their receptors.

The set so defined has numerous clusters of sequence-related ligands and receptors. Such clusters are likely to have arisen from tandem gene duplications, and thus individual genes cannot be treated as independently positioned. To eliminate the effects of tandem duplication we perform an all versus all blast (with E < 0.01) of the coding sequences defined from the RefSeqs for each gene. For each pair of putative duplicates on the same chromosome one of the two was randomly selected to be removed. In a tandem cluster with more than two duplicates only one gene was considered. Using E < 0.2 resolves to the same data set.

With this approach, however, a few well described duplicate clusters are not identified. For example, there remain 6 ligand-receptor pairs associated with the 2q14 cluster of three ligands (IL1 alpha, beta and the receptor antagonist) and their two receptors (IL1R1 and Il1R2) in 2q12. However, while Blast fails to reveal either of these clusters as duplicate clusters, this contradicts the conventional wisdom, based on close analysis of gene structure, function and conserved functional parts, that they are both duplicate arrays [51,52]. The problem in this instance is most probably that interleukins and their receptors tend often to be fast evolving, hence liable to avoid detection as duplicates unless they are relatively modern duplicates. Indeed this inability of Blast to identify orthology/paralogy of fast evolving genes has recently been well demonstrated [53].

To eliminate such problems we additionally remove one of a pair of ligands within 1 Mb of each other. Likewise we remove one of a pair of receptors should the receptors occur within 1 Mb of each other. In nearly all cases the ligands in the cluster also bind the same receptors and vice versa. One exception is Insulin-like growth factor 2 (*Igf2*) and Insulin, which, while sequence related and very closely linked, bind different receptors. In both cases the receptors are unlinked. Given the sequence relatedness we remove one of the two. In effect we are then asking about a tendency for a ligand cluster to be linked to a receptor cluster. The final data set specifies 108 ligand-receptor sets (106 in the non-augmented set) [see Additional file 1]. Note that most ligands have more than one receptor. When then we refer to ligand receptor "pairs," we refer to incidence in which a ligand is linked to one of its receptors. We also performed the same analyses as given below on a data set in which duplicates are defined exclusively by reference to Blast scores [see Additional file 2]. A list of linked ligands and receptors in this data are provided [see Additional file 3]. Using both data sets, in both augmented and non-augmented form (i.e. with or without the two ligand-receptor pairs highlighted by Copper (1999)) we obtain qualitatively identical results [see Additional file 4].

### Mouse data

For analysis of the patterns in mice we identified the orthologs of the human ligands and receptors by reference to the MGI curated set of mouse-human orthologs [54]. For each human gene, the locus link id was cross referenced to the mouse ortholog. Thirteen genes lacking an ortholog were removed. Mouse locus link numbers were employed to access RefSeq numbers, unigene references and the chromosomal locations. Breadth of expression was derived from Unigene cDNA source annotations. The compilation of all mouse genes and their position used in the randomization was derived from MartView [55] at Ensembl requesting those with described LocusLink ids. The positions of the ligands and receptors were found by cross-referencing their LocusLink ids to this Ensembl data set. The few that failed to be resolved by this method were ascribed a position by Blasting their RefSeq against the complete mouse genome [55]. For the randomizations only those genes with well resolved genomic locations were employed (24742 genes).

### Randomization and statistics

To ask whether there are more ligand-receptor pairs on the same chromosome than expected by chance, we calculate the observed number and compare this with simulants in which we randomly permute the positions of all ligands and receptors. It is unclear on *a priori *grounds, however, what should be the null model for the randomization. We consider three possible models.

First we suppose that a ligand or receptor can occur in any location in the genome currently occupied by a protein coding gene. In this instance, in the human genome, the positions permitted in the randomizations correspond to the annotated positions of the 24,300 protein coding genes in the NC_0000n files for the human genome (n from 01–23).

Second, we assume that a ligand or receptor can occur in any location in the genome currently occupied by a protein coding gene with the same or comparable expression breadth. For each of the genes in the complete human and mouse sets we identified the Unigene id by following the LocusLink page pertinent to each gene and identified the breadth of expression in the same way as for the ligand-receptor set. Genes were placed in bins of 0–4, 5–9 tissues etc in which they were expressed. We consider two bin classification systems. In both, ligands and receptors in these randomizations were permitted to reside in the same location as any gene in the same bin from the complete human set.

Third, we suppose that there is something unique about ligands and receptors, such that each ligand or receptor can only be relocated to the position of another ligand or receptor. Breadth of expression is ignored as this too greatly constrains the randomizations.

For each of the above randomization protocols we determined the number of ligand-receptor pairs on the same chromosome. Significance (*P*) was determined from *P *= (*r*+1)/(*n*+1), where *r *is the number of simulants with the same or greater number of ligand-receptor pairs than observed in the real data and *n *is the number of simulants (10,000 in all instances), this being the unbiased estimator [56,57].

To determine whether the ligand-receptor pairs that we observe on a given chromosome are more closely linked than expected we perform two analogous sets of simulations. In the first we calculate the mean distance between these pairs and compare this with the mean of the simulants described above. In the second we ask about the number of ligand-receptor pairs within a given distance of each other (e.g., within 1 Mb). We then compare this number to the mean number found after permuting all genes within the chromosomes within which they are found. By permuting on the same chromosome we control for the number of ligand-receptor pairs on the same chromosome.

All results prove to be insensitive to which of the three randomization null models is employed. Unless stated otherwise a result in the text relates to the first model in the text. All results can be found in attached files, for humans [see Additional file 4] and for mice [see Additional file 5] [see Additional file 6].

## Abbreviations

Mb: megabase

Df: degrees of freedom

## Authors' contributions

Both authors contributed to analysis, design and write up.

## Supplementary Material

Additional file 4 **Supplement 4: **The results and analysis of randomizations, excluding randomizations controlling for breadth. The results sheet is split in two. The top half ("all genes") refers to analysis of the augmented ligand receptor data set, i.e. the one containing the two ligand-receptor pairs nominated by Cooper. The lower half excludes these two. In each, as detailed in the methods, two filtering methods were used to eliminate tandem duplicates: Blast alone (see Additional file 2) and blast plus close proximity of ligands, close proximity of receptors (see Additional file 1). Within each of these subdivisions two data sets were employed to determine where, in randomizations, a ligand and/or receptor might locate. In one case (lig/rec genes only), the positions of ligands and receptors were interchanged with ligands/receptors derived from the test set. In the second method, possible locations are anywhere in the genome where a gene, of any variety, resides ("all genes in genome"). Randomizations for each of the analysis in turn was performed either permitting a ligand or receptor to locate anywhere in the genome ("all genes") or anywhere on the same chromosome ("within chr"). Analysis was further performed on those ligand receptor sets in which at least one of the genes in the set was placentally expressed ("placental"). N is the number of genes in the test set, "sameChr", refers to the number of incidences of ligand receptor pairs on the same chromosome, "sameChrRand"", is the corresponding number for the randomizations. P_chr is the P value for the comparisons. <d> is the mean distance between ligands and receptors on the same chromosome (in kb) and <dRand> the corresponding number in the randomizations. P_d is the relevant P value. "Count[Nkb]", "countRand, P refer to the number of incidences in the real data set of ligand receptor pairs less than N kb apart, the number in the randomizations and the P value. This statistic is only relevant for the cases where randomization is done within chromosomes. The between chromosome randomization by its nature confounds the effect of the excess of pairs on the same chromosome as well as their proximity. Click here for file

Additional file 5 **Supplement 5: **This supplement details the analysis (without control for breadth of expression) in the mouse data sets. Click here for file

Additional file 6 **Supplement 6: **This supplement details the analysis with control for breadth of expression in the mouse. Click here for file

Additional file 7 **Supplement 7: **This supplement details the evidence for co-duplication for paralogous ligand-receptor pairs Click here for file

Additional file 1 **Supplement 1. **The data set of ligand receptor pairs after purging of tandem duplicates by Blast score and by purging of ligands within 1 Mb of each other and receptors within 1 MB of each other. Unigene_id is the Unigene reference number. Entrez_id is the Entrez/LocusLink number. Chr is the chromosome on which the gene is found. Cytog is the cytogenetic location of the gene. Genbank_chr_file is the relevant accession number of the whole chromosome GenBank file. From, to, midpos are the ends of the gene and the middle position of the gene in base pairs, these referring to the locations in the NC files. Number of tissues is a count of the number of entries in cDNA source entry in the Unigene page. Placental: 1 means placentally expressed, 0 means no evidence for placental expression. GI: GI number. RefSeq: the ReqSeq Genbank number Click here for file

Additional file 2 **Supplement 2: **The data set of ligand receptor pairs after purging of tandem duplicates by Blast scores alone. Annotation as above. Click here for file

Additional file 3 **Supplement 3: **The set of ligand-receptor pairs on the same chromosome in the data set given in supplement 2 (Blast score purged alone). Annotation as for figure 1 in the table. Note: In the above three data sets the final two ligand-receptor pairs are those nominated by Cooper. Click here for file

## References

[B1] Hurst LD, Pal C, Lercher MJ (2004). The evolutionary dynamics of eukaryotic gene order. Nature reviews Genetics.

[B2] Caron H, van Schaik B, van der Mee M, Baas F, Riggins G, van Sluis P, Hermus MC, van Asperen R, Boon K, Voute PA, Heisterkamp S, van Kampen A, Versteeg R (2001). The human transcriptome map: Clustering of highly expressed genes in chromosomal domains. Science.

[B3] Lercher MJ, Urrutia AO, Hurst LD (2002). Clustering of housekeeping genes provides a unified model of gene order in the human genome. Nature Genetics.

[B4] Lercher MJ, Urrutia AO, Pavlicek A, Hurst LD (2003). A unification of mosaic structures in the human genome. Hum Mol Genet.

[B5] Versteeg R, van Schaik BDC, van Batenburg MF, Roos M, Monajemi R, Caron H, Bussemaker HJ, van Kampen AHC (2003). The human transcriptome map reveals extremes in gene density, intron length, GC content, and repeat pattern for domains of highly and weakly expressed genes. Genome Res.

[B6] Cohen BA, Mitra RD, Hughes JD, Church GM (2000). A computational analysis of whole-genome expression data reveals chromosomal domains of gene expression. Nature Genet.

[B7] Hurst LD, Williams EJ, Pal C (2002). Natural selection promotes the conservation of linkage of co-expressed genes. Trends Genet.

[B8] Singer GAC, Lloyd AT, Huminiecki LB, Wolfe KH (2005). Clusters of co-expressed genes in mammalian genomes are conserved by natural selection. Mol Biol Evol.

[B9] Lercher MJ, Blumenthal T, Hurst LD (2003). Coexpression of neighboring genes in Caenorhabditis elegans is mostly due to operons and duplicate genes. Genome Res.

[B10] Roy PJ, Stuart JM, Lund J, Kim SK (2002). Chromosomal clustering of muscle-expressed genes in Caenorhabditis elegans. Nature.

[B11] Miller MA, Cutter AD, Yamamoto I, Ward S, Greenstein D (2004). Clustered organization of reproductive genes in the C. elegans genome. Curr Biol.

[B12] Spellman PT, Rubin GM (2002). Evidence for large domains of similarly expressed genes in the Drosophila genome.. J Biol.

[B13] Boutanaev AM, Kalmykova AI, Shevelyou YY, Nurminsky DI (2002). Large clusters of co-expressed genes in the Drosophila genome. Nature.

[B14] Thygesen H, Zwinderman A (2005). Modelling the correlation between the activities of adjacent genes in drosophila. BMC Bioinformatics.

[B15] Williams EJB, Bowles DJ (2004). Coexpression of neighboring genes in the genome of Arabidopsis thaliana. Genome Res.

[B16] Lee JM, Sonnhammer EL (2003). Genomic gene clustering analysis of pathways in eukaryotes. Genome Res.

[B17] Wong S, Wolfe KH (2005). Birth of a metabolic gene cluster in yeast by adaptive gene relocation. Nature Genet.

[B18] Fukuoka Y, Inaoka H, Kohane IS (2004). Inter-species differences of co-expression of neighboring genes in eukaryotic genomes. BMC Genomics.

[B19] Teichmann SA, Veitia RA (2004). Genes encoding subunits of stable complexes are clustered on the yeast chromosomes: An interpretation from a dosage balance perspective. Genetics.

[B20] Cooper DN (1999). Human Gene Evolution.

[B21] Lennard A, Gorman P, Carrier M, Griffiths S, Scotney H, Sheer D, Solari R (1992). Cloning and chromosome mapping of the human interleukin-1 receptor antagonist gene. Cytokine.

[B22] Wang MH, Ronsin C, Gesnel MC, Coupey L, Skeel A, Leonard EJ, Breathnach R (1994). Identification of the Ron gene product as the receptor for the human macrophage stimulating protein. Science.

[B23] Popovici C, Leveugle M, Birnbaum D, Coulier F (2001). Coparalogy: Physical and functional clusterings in the human genome. Biochem Biophys Res Commun.

[B24] Haig D (1996). Gestational drive and the green-bearded placenta. Proc Nat Acad Sci USA.

[B25] Peters LL, Barker JE (1993). Novel inheritance of the murine severe combined anemia and thrombocytopenia (Scat) phenotype.. Cell.

[B26] Hurst LD (1993). scat+  is a selfish gene analogous to Medea of Tribolium castaneum. Cell.

[B27] Fisher RA (1930). The Genetical Theory of Natural Selection.

[B28] Bodmer WF, Parsons PA (1962). Linkage and recombination in evolution. Adv Genet.

[B29] Nei M (1967). Modification of linkage intensity by natural selection. Genetics.

[B30] Nei M (1968). Evolutionary change in linkage intensity. Nature.

[B31] Marquardt T, Shirasaki R, Ghosh S, Andrews SE, Carter N, Hunter T, Pfaff SL (2005). Coexpressed EphA receptors and Ephrin-A iigands mediate opposing actions on growth cone navigation from distinct membrane domains. Cell.

[B32] Graeber TG, Eisenberg D (2001). Bioinformatic identification of potential autocrine signaling loops in cancers from gene expression profiles. Nat Genet.

[B33] DLRP database. http://dip.doe-mbi.ucla.edu/files/dlrp/dlrp.txt.

[B34] Keightley PD, Lercher MJ, Eyre-Walker A (2005). Evidence for widespread degradation of gene control regions in hominid genomes. PLoS Biol.

[B35] Keightley PD, Eyre-Walker A (2000). Deleterious mutations and the evolution of sex. Science.

[B36] Duret L, Mouchiroud D, Gouy M (1994). HOVERGEN - a database of homologous vertebrate genes. Nucl Acids Res.

[B37] McLysaght A, Hokamp K, Wolfe KH (2002). Extensive genomic duplication during early chordate evolution. Nat Genet.

[B38] Pebusque MJ, Coulier F, Birnbaum D, Pontarotti P (1998). Ancient large-scale genome duplications: phylogenetic and linkage analyses shed light on chordate genome evolution. Mol Biol Evol.

[B39] Katsanis N, Fitzgibbon J, Fisher EMC (1996). Paralogy Mapping: Identification of a Region in the Human MHC Triplicated onto Human Chromosomes 1 and 9 Allows the Prediction and Isolation of NovelPBXandNOTCHLoci. Genomics.

[B40] Stankiewicz P, Shaw CJ, Withers M, Inoue K, Lupski JR (2004). Serial segmental duplications during primate evolution result in complex human genome architecture. Genome Res.

[B41] Veitia RA (2002). Exploring the etiology of haploinsufficiency. Bioessays.

[B42] Veitia RA (2005). Gene dosage balance: deletions, duplications and dominance. Trends Genet.

[B43] Papp B, Pal C, Hurst LD (2003). Dosage sensitivity and the evolution of gene families in yeast. Nature.

[B44] Bourque G, Zdobnov EM, Bork P, Pevzner PA, Tesler G (2005). Comparative architectures of mammalian and chicken genomes reveal highly variable rates of genomic rearrangements across different lineages. Genome Res.

[B45] Zhao SY, Shetty J, Hou LH, Delcher A, Zhu BL, Osoegawa K, de Jong P, Nierman WC, Strausberg RL, Fraser CM (2004). Human, mouse, and rat genome large-scale rearrangements: Stability versus speciation. Genome Res.

[B46] O'Brien SJ, Menotti-Raymond M, Murphy WJ, Nash WG, Wienberg J, Stanyon R, Copeland NG, Jenkins NA, Womack JE, Graves JAM (1999). The promise of comparative genomics in mammals. Science.

[B47] Fares MA, Byrne KP, Wolfe KH (2005). Rate Asymmetry After Genome Duplication Causes Substantial Long Branch Attraction Artifacts in the Phylogeny of Saccharomyces Species. Mol Biol Evol.

[B48] Popovici C, Roubin R, Coulier F, Birnbaum D (2005). An evolutionary history of the FGF superfamily. Bioessays.

[B49] Unigene. http://www.ncbi.nlm.nih.gov/entrez/query.fcgi?db=unigene.

[B50] Entrez. http://www.ncbi.nlm.nih.gov/entrez/query.fcgi?CMD=search&DB=gene.

[B51] Steinkasserer A, Spurr NK, Cox S, Jeggo P, Sim RB (1992). The human IL-1 receptor antagonist gene (IL1RN) maps to chromosome 2q14-q21, in the region of the IL-1 alpha and IL-1 beta loci. Genomics.

[B52] Dale M, Nicklin MJ (1999). Interleukin-1 receptor cluster: gene organization of IL1R2, IL1R1, IL1RL2 (IL-1Rrp2), IL1RL1 (T1/ST2), and IL18R1 (IL-1Rrp) on human chromosome 2q. Genomics.

[B53] Wolfe K (2004). Evolutionary genomics: yeasts accelerate beyond BLAST. Curr Biol.

[B54] MGD Human Mouse orthologs. ftp://ftp.informatics.jax.org/pub/reports/HMD_Human4.rpt.

[B55] Ensembl MartView. http://www.ensembl.org/Multi/martview?species=Mus_musculus.

[B56] Davison AC, Hinkley DV (1997). Bootstrap methods and their application.

[B57] North BV, Curtis D, Sham PC (2002). A note on the calculation of empirical P values from Monte Carlo procedures. Am J Hum Genet.

[B58] Jin M, Chen Y, He S, Ryan SJ, Hinton DR (2004). Hepatocyte growth factor and its role in the pathogenesis of retinal detachment. Invest Ophthalmol Vis Sci.

[B59] Schmidt L, Duh FM, Chen F, Kishida T, Glenn G, Choyke P, Scherer SW, Zhuang Z, Lubensky I, Dean M, Allikmets R, Chidambaram A, Bergerheim UR, Feltis JT, Casadevall C, Zamarron A, Bernues M, Richard S, Lips CJ, Walther MM, Tsui LC, Geil L, Orcutt ML, Stackhouse T, Zbar B (1997). Germline and somatic mutations in the tyrosine kinase domain of the MET proto-oncogene in papillary renal carcinomas. Nat Genet.

[B60] Bezerra JA, Carrick TL, Degen JL, Witte D, Degen SJ (1998). Biological effects of targeted inactivation of hepatocyte growth factor-like protein in mice. J Clin Invest.

[B61] Muraoka RS, Sun WY, Colbert MC, Waltz SE, Witte DP, Degen JL, Friezner Degen SJ (1999). The Ron/STK receptor tyrosine kinase is essential for peri-implantation development in the mouse. J Clin Invest.

[B62] Joutel A, Corpechot C, Ducros A, Vahedi K, Chabriat H, Mouton P, Alamowitch S, Domenga V, Cecillion M, Marechal E, Maciazek J, Vayssiere C, Cruaud C, Cabanis EA, Ruchoux MM, Weissenbach J, Bach JF, Bousser MG, Tournier-Lasserve E (1996). Notch3 mutations in CADASIL, a hereditary adult-onset condition causing stroke and dementia. Nature.

[B63] Miller DL, Ortega S, Bashayan O, Basch R, Basilico C (2000). Compensation by fibroblast growth factor 1 (FGF1) does not account for the mild phenotypic defects observed in FGF2 null mice. Mol Cell Biol.

[B64] Kawaguchi H, Nakamura K, Tabata Y, Ikada Y, Aoyama I, Anzai J, Nakamura T, Hiyama Y, Tamura M (2001). Acceleration of Fracture Healing in Nonhuman Primates by Fibroblast Growth Factor-2. J Clin Endocrinol Metab.

[B65] Weinstein M, Xu X, Ohyama K, Deng CX (1998). FGFR-3 and FGFR-4 function cooperatively to direct alveogenesis in the murine lung. Development.

[B66] Zammit C, Coope R, Gomm JJ, Shousha S, Johnston CL, Coombes RC (2002). Fibroblast growth factor 8 is expressed at higher levels in lactating human breast and in breast cancer. Br J Cancer.

[B67] Muenke M, Schell U, Hehr A, Robin NH, Losken HW, Schinzel A, Pulleyn LJ, Rutland P, Reardon W, Malcolm S (1994). A common mutation in the fibroblast growth factor receptor 1 gene in Pfeiffer syndrome. Nat Genet.

[B68] Xu J, Liu Z, Ornitz DM (2000). Temporal and spatial gradients of Fgf8 and Fgf17 regulate proliferation and differentiation of midline cerebellar structures. Development.

[B69] Hu MC, Qiu WR, Wang YP, Hill D, Ring BD, Scully S, Bolon B, DeRose M, Luethy R, Simonet WS, Arakawa T, Danilenko DM (1998). FGF-18, a novel member of the fibroblast growth factor family, stimulates hepatic and intestinal proliferation. Mol Cell Biol.

[B70] OMIM. http://www.ncbi.nlm.nih.gov/entrez/query.fcgi?db=OMIM.

