# Supplementary material for: Unusual linkage patterns of ligands and their cognate receptors indicate a novel reason for non-random gene order in the human genome
Source: BMC Evol Biol. 2005 Nov 8;5:62. doi: 10.1186/1471-2148-5-62 (PMC1309615; doi:10.1186/1471-2148-5-62)
Supplement: Additional file 7 — Supplement 7: This supplement details the evidence for co-duplication for paralogous ligand-receptor pairs [file 1471-2148-5-62-S7.doc]

**Evidence for co-duplication for paralogous ligand-receptor pairs**

The linkage of the FGFs to their receptors has been previously noted [1] and been argued, from phylogenetics data, to be owing to block or whole genome duplications [1]. However, linkage of both FGF1 and FGF18 with FGFR4 on human chromosome 5, probably reflects a very ancient duplication of the ligands to produce the two growth factors (FGF1 and FGF18) linked to an ancestral receptor [1]. The duplication of this cluster (two ligands one receptor) would, it is proposed[1], have resulted in the FGF2/FGFR3 linkage along with linkage of FGF24 (the duplicate of FGF18), which is also in linkage with FGFR3. The other pairs (FGF8/FGFR2; FGF17, FGFR1) are in turn proposed to also be duplications of the same sets of genes [1].

Inspection of the phylogenetic tree of the FGFR family as presented in Hovergen [2], including urochordate data, suggests that at the base of the vertebrates there was one receptor which duplicated to produce the ancestors of FGFR1/2 and FGFR3/4. Duplication of both ancestral sequences then occurred very shortly after (prior to the divergence of the fish), leaving FGFR1 and 2 as nearest paralogs and FRGR3 and FGFR4 as nearest paralogs. If there was co-duplication of the receptors we should then expect to see FGF1 and FGF2 as nearest paralogs and FRF8 and FGF17 as nearest paralogs, with, in both incidences, duplication occurring near the base of the vertebrates. The nearest paralog relationships are upheld [1]. In both instances the duplication occurred prior to the divergence of fish but whether it occurred post the urochordates is unknown owing to a paucity of relevant data. Whether this was via whole genome duplication or smaller scale block duplication has yet to be resolved.

As regards DLL1/DLL3 with NOTCH3/NOTCH4 we are unaware of any evidence to say that, in these two cases, the ancestral duplication held both the ligand and the receptor, although it is notable that *Notch* 1,3 and4 are linked to cognate receptors in the mouse genome as well, as is *Fgfr2* (see table 4). Phylogenies [2] of Het/Mst1 and Met/MSTR1 both suggest duplication occurred before the fish divergence (i.e. prior to or at the base of the vertebrates). In neither case, however, could we identify invertebrate othologs in complete genomes (fly and worm). With absence of good invertebrate data, these results are not definitive.

1. Popovici C, Roubin R, Coulier F, Birnbaum D: **An evolutionary history of the FGF superfamily**. *Bioessays* 2005, **27**(8):849-857.

2. Duret L, Mouchiroud D, Gouy M: **HOVERGEN - a database of homologous vertebrate genes**. *Nucl Acid Res* 1994, **22**:2360-2365.
